# Draft Genome Sequence of Porphyromonas gingivalis Strain 381 Okayama (381OKJP) Stock Culture

**DOI:** 10.1128/MRA.01641-18

**Published:** 2019-02-28

**Authors:** Anthony C. May, Hiroshi Maeda, Hidemi Kurihara, Manabu Miyamoto, Hiroshi Hongyo, Ichiro Tanimoto, Atsushi Nagai, Fusanori Nishimura, Yoji Murayama, Keijiro Kato, Susumu Kokeguchi, Carla Cugini

**Affiliations:** a Department of Oral Biology, Rutgers School of Dental Medicine, Newark, New Jersey, USA; b Department of Endodontics, Osaka Dental University, Hirakata, Osaka, Japan; c Department of Periodontal Medicine, Graduate School of Biomedical & Health Sciences, Hiroshima University, Hiroshima, Japan; d Miyamoto Dental & Orthodontic Office, Kobe, Hyogo, Japan; e Tanpopo Dental Clinic, Soja, Okayama, Japan; f National Sanatorium Oshima Seisho-en, Takamatsu, Kagawa, Japan; g Regional Liaison Center, Fukuoka Dental College, Fukuoka, Japan; h Section of Periodontology, Division of Oral Rehabilitation, Kyushu University Faculty of Dental Science, Fukuoka, Japan; i Department of Pathophysiology-Periodontal Science, Graduate School of Medicine, Dentistry and Pharmaceutical Sciences, Okayama University, Okayama, Japan; j Department of Oral Microbiology, Graduate School of Medicine, Dentistry and Pharmaceutical Sciences, Okayama University, Okayama, Japan; University of Maryland School of Medicine

## Abstract

We report the draft genome sequence of Porphyromonas gingivalis strain 381 Okayama (381OKJP). The strain, obtained from the Socransky collection, has been used for experimentation since 1987. This sequence allows for comparisons to other sequenced 381 strains to observe acquisition of mutations and genome rearrangements in a commonly used laboratory strain.

## Abstract (precis)

We report the draft genome sequence of Porphyromonas gingivalis strain 381 Okayama (381OKJP). The strain, obtained from the Socransky collection, has been used for experimentation since 1987.

## ANNOUNCEMENT

The Gram-negative anaerobe Porphyromonas gingivalis is recognized as a key oral pathobiont associated with human periodontitis. P. gingivalis strain 381, isolated at the Forsyth Institute (ca. 1970s), is a globally distributed legacy strain extensively used in oral microbiology research. Strain 381OKJP, stored in Japan for ∼40 years, displays location-dependent genetic and phenotypic variability, likely due to interlaboratory exchange (1–8). Recently, the Progulske-Fox group reported the genome sequence for strain 381, which based on genome cluster analysis and gene order revealed the strain to be closely related to ATCC 33277 (9). Strain 381OKJP, selected for this analysis, a generous gift from Sigmund S. Socransky (ca. 1987), was originally deposited in the culture collection at the Department of Oral Microbiology at the Okayama University Dental School (10). Strain 381OKJP (*fimA* genotype V, *mfa1* genotype II [53-kDa Mfa1], IS*Pg4*) is phenotypically distinct from the previously reported strain 381 (*fimA* genotype I, *mfa1* genotype I [67/75-kDa Mfa1], no IS*Pg4*). This study confirms the worldwide distribution of variable 381 strains. The genome sequence of 381OKJP will provide for strain comparisons and analyses of genome rearrangements and information related to 381OKJP-specific phenotypic characteristics.

P. gingivalis 381OKJP was cultured in duplicate at 37°C in Gifu anaerobic medium (GAM) broth, modified (Nissui Pharmaceutical Co., Japan), containing 10 μg/ml hemin and 5 μg/ml vitamin K_1_ under anaerobic conditions (<0.1% oxygen, >15% CO_2_) in a GasPak 100 jar (Becton, Dickinson, USA) with an AnaeroPack-Anaero generator (Mitsubishi Gas Chemical Company, Japan). Cells were collected by centrifugation and washed twice with Gibco phosphate-buffered saline (Thermo Fisher Scientific, Japan). Genomic DNA from two independent samples was extracted and purified using a NucleoSpin tissue kit (TaKaRa Bio, Japan), according to the manufacturer’s instructions.

Genomic libraries containing 150- to 550-bp inserts were constructed using the Kapa HyperPlus library kit. The duplicate libraries were independently paired-end sequenced using the Illumina MiSeq platform, and run statistics were determined using CLC Genomics Workbench (version 9.0.1; CLC Bio) (Table 1). The A5-miseq assembly pipeline was used to automate read trimming and adapter removal from the paired-end FASTQ data and to assemble the reads into contigs (version 20160826) (11, 12). QUAST was used to check the quality of each assembly and compare contigs to previously published genomes available from the NCBI (13). The independent genomes were aligned against each other with progressiveMauve, and the Mauve Contig Mover was used iteratively to infer contig order (version 2.4.0 [14]). The final draft genome reported here contains 1,296,214 reads (*N*_50_, 44,243 bp), producing 128 contigs with 75.89-fold coverage for error-corrected bases.

**TABLE 1:** Run statistics for duplicate *P. gingivalis* 381OKJP samples

Characteristic by *P. gingivalis* 381OKJP sample	No.	Avg length (bp)	Total no. of bases
Sample 1			
Reads	982,843	100.75	99,023,010
Matched reads	781,573	121.95	95,309,470
Unmatched reads	201,270	18.45	3,713,540
Contigs	114	20,023	2,282,631
Reads in pairs	271,650	312.7	
Broken paired reads	203,320	100	
Sample 2			
Reads	1,041,029	94.8	98,689,508
Matched reads	802,956	118.62	95,242,682
Unmatched reads	238,073	14.48	3,446,826
Contigs	109	20,967	2,285,486
Reads in pairs	246,768	312.91	
Broken paired reads	234,307	101.13	

The final assembly resulted in 2,331,065 bp, with a GC content of 48.4%, consistent with NBCI-deposited P. gingivalis genomes. To confirm taxonomic classification and identify lateral gene transfer events, all scaffolds were aligned to the nonredundant-microbial_20140513 reference database using the LAST algorithm with the Taxator toolkit (v1.3.3e [15]; v938 [16]). Taxonomic classification was supported by 1,720,921 bp, and all assigned sample sequence segments were homologous to those of P. gingivalis. Annotation was performed by the NCBI using the Prokaryotic Genome Annotation Pipeline (PGAP; best-placed reference protein, version 4.2 [17]).

### Data availability.

This whole-genome shotgun project has been deposited at DDBJ/ENA/GenBank under the accession number QPGS00000000. The version described in this paper is version QPGS01000000. The raw reads have been deposited at the NCBI/SRA under BioProject number PRJNA475798.
